# When signalling goes wrong: pathogenic variants in structural and signalling proteins causing cardiomyopathies

**DOI:** 10.1007/s10974-017-9487-3

**Published:** 2017-11-08

**Authors:** Mehroz Ehsan, He Jiang, Kate L.Thomson, Katja Gehmlich

**Affiliations:** Division of Cardiovascular Medicine, Radcliffe Department of Medicine and British Heart Foundation Centre of Research Excellence, University of Oxford, Oxford, UK

**Keywords:** Cardiomyopathies, Genetic pathogenic variant, Mutation, Variant of unknown significance, Signalling, Titin, Mouse models, Heart

## Abstract

Cardiomyopathies are a diverse group of cardiac disorders with distinct phenotypes, depending on the proteins and pathways affected. A substantial proportion of cardiomyopathies are inherited and those will be the focus of this review article. With the wide application of high-throughput sequencing in the practice of clinical genetics, the roles of novel genes in cardiomyopathies are recognised. Here, we focus on a subgroup of cardiomyopathy genes [*TTN, FHL1, CSRP3, FLNC* and *PLN*, coding for Titin, Four and a Half LIM domain 1, Muscle LIM Protein, Filamin C and Phospholamban, respectively], which, despite their diverse biological functions, all have important signalling functions in the heart, suggesting that disturbances in signalling networks can contribute to cardiomyopathies.

## Introduction

Inherited cardiomyopathies (CMs) are genetic diseases of the heart; the majority of them are inherited in an autosomal-dominant (AD) pattern. These diseases can be classified primarily on the basis of dominating morphological and/or functional changes observed in the heart muscle of the affected individual. The different types include dilated cardiomyopathy (DCM), hypertrophic cardiomyopathy (HCM), restrictive cardiomyopathy (RCM), arrhythmogenic right ventricular cardiomyopathy (ARVC) and left ventricular non-compaction (LVNC) cardiomyopathy (reviewed in Watkins et al. 2011; Yacoub 2014). Many CMs are also associated with life-threatening arrhythmias (reviewed in Bezzina et al. 2015), that potentially result in sudden cardiac death events, making the identification and risk stratification of patients an important issue in the clinical practice.

Despite their different morphological appearances there is significant overlap of the underlying molecular pathways among various CMs. For example, cellular hypertrophy of cardiomyocytes is commonly observed in both DCM and HCM (Davis et al. 2016). Apoptosis, leading to myocyte death, is a prominent feature of DCM and ARVC (Narula et al. 1996; Thiene et al. 1997). Additionally, fibrosis, caused by fibroblast activation and collagen deposition—often in response to apoptosis of cardiomyocytes—is common among DCM, ARVC and HCM (Burlew and Weber 2000).

The genetic aetiology of cardiomyopathies is best understood for HCM, DCM and ARVC. Historically, genomic-wide linkage analysis in large families led to the identification of the first HCM disease gene loci (Solomon et al. 1990; Thierfelder et al. 1993; Watkins et al. 1993). The identification of de novo pathogenic variants in *MYH7*, the gene encoding sarcomeric beta (β)-Myosin heavy chain, confirmed it as causal gene in HCM (Watkins et al. 1992, 1995). Together with the discovery of pathogenic variants in *TPM1* and *TNNT2* (encoding the thin filament proteins Tropomyosin and Troponin T) and *MYBPC3* (encoding Myosin Binding Protein C), the paradigm of HCM as a “disease of the sarcomere” was postulated (Geisterfer-Lowrance et al. 1990; Thierfelder et al. 1994).

DCM is a more clinically heterogeneous condition. The vast majority of cases have a “non-genetic” aetiology (e.g. ischemic heart disease, viral myocarditis, substance abuse). However, in individuals with so called idiopathic DCM, in whom the common “non-genetic” causes have been excluded, approximately a quarter of cases appear to be familial (Petretta et al. 2011).

Over 50 genes have been reported to cause DCM, either as an isolated phenotype, or as part of a syndrome. These genes encode proteins with a diverse range of structural and functional roles within the cardiac myocyte (e.g. sarcomere, nuclear membrane, desmosome, sarcoplasmic reticulum, cytoskeleton). The majority of non-syndromic DCM is inherited in an AD manner; however autosomal recessive and X-linked forms are also reported (Hershberger et al. 2013; McNally et al. 2013).

In individuals with AD non-syndromic DCM, loss-of-function variants in the *TTN* gene, which encodes the protein Titin, are the most commonly reported genetic defect (Herman et al. 2012; Pugh et al. 2014; Walsh et al. 2017) and will be discussed below.

Pathogenic variants in *LMNA* (encoding the nuclear membrane protein isoforms Lamin A and Lamin C), and *MYH7* appear to be the second most common, accounting for between 4–6 and 4–5% of cases respectively (Haas et al. 2015; Pugh et al. 2014; Walsh et al. 2017).

Pathogenic variants in many other genes, including protein components of the sarcomere (e.g. *TNNT2, TPM1*), Z-disk (e.g. *TCAP, MYPN, NEXN*), cytoskeleton (e.g. *DES, VCL*), desmosome (e.g. *DSP*), and RNA-binding proteins (e.g. *RBM20*), have been reported in DCM cohorts. Individually, these genes appear to account for a smaller proportion of cases (Haas et al. 2015; Pugh et al. 2014; Walsh et al. 2017).

ARVC is recognised as a “disorder of the desmosome”, due to the majority of causal variants arising in genes encoding proteins in this cell–cell contact structure (e.g. *PKP2, DSG2, DSC2, DSP* and *JUP*) (Awad et al. 2008).

In the recent years, substantial advances have been made in our understanding of genetic causes of cardiomyopathies through the application of high-throughput genetic sequencing techniques. Genomic sequencing in large reference cohorts has revealed unexpectedly high levels of rare variation in cardiomyopathy genes in the background population (Andreasen et al. 2013; Walsh et al. 2017). Simultaneously, it has become feasible to analyse more candidate genes in larger patient cohorts, and to explore genes which, due to their large size, were technically difficult to analyse (e.g. *TTN, DMD* and *RYR2*). This has facilitated the identification of novel disease genes, and enabled re-evaluation of existing gene–disease relationships.

The current major challenge in cardiomyopathy gene analysis is variant interpretation; in many cardiomyopathy disease genes, it is difficult to distinguish between disease-causing and benign variation. Demonstrating the lack of suitable approaches beyond bioinformatics prediction tools, an increasing proportion of variants—especially missense changes—are being classified as “variants of unknown significance” (Alfares et al. 2015; Pugh et al. 2014; Waldmuller et al. 2015; Walsh et al. 2017). Insights into the detailed molecular mechanisms of disease are another challenging aspect of cardiomyopathies and usually lack behind the genetic discoveries.

**Fig. 1:**
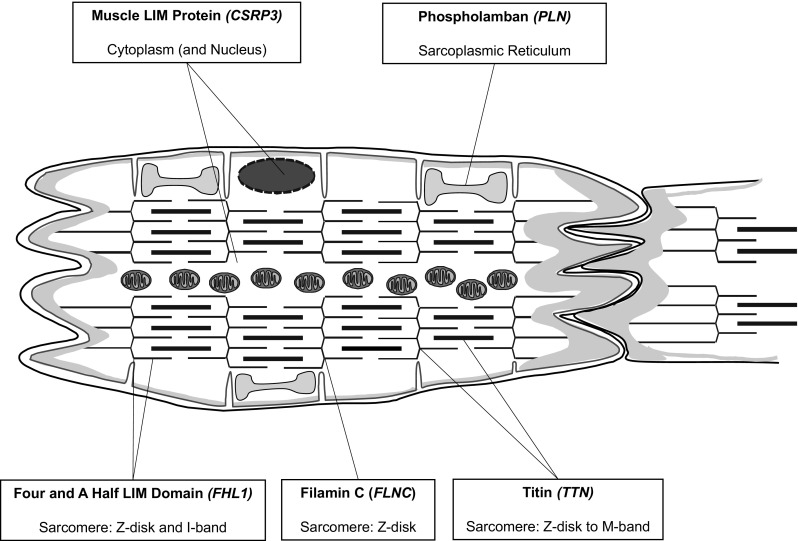
Schematic localisation of the five proteins of this review in a drawing of a cardiomyocyte; genes name are given in brackets; adapted from Cahill and Gehmlich 2015 with permission

In this review we discuss selected examples of cardiomyopathy genes (*TTN, FHL1, CSRP3, FLNC* and *PLN*; see Table 1 and Fig. 1) which, based on their known biological functions and the (limited) functional work on the disease-causing pathogenic variants, have been shown to have important signalling functions in the heart. It is proposed that perturbations of these signalling functions in the presence of pathogenic genetic variants can cause cardiomyopathy.

**Table 1:** Summary of cardiac diseases caused by pathogenic variants in *TTN, FHL1, CSRP3, FLNC* and *PLN*

Gene/chromosome	Disease	Inheritance pattern	Comments
2q31.2 *TTN*	DCM	AD, variable penetrance	Truncating variants in A-band dominating, common (≤25%)
Xq26.3 *FHL1*	HCM	X-linked	With or without skeletal muscle involvement, rare
11p15.1 *CSRP3*	HCM	AD, late onset	Rare; missense variants dominating
*FLNC* 7q32.1	HCM DCM	AD AD	Missense variants dominating Truncating variants dominating
*PLN* 6q22.31	DCM HCM	AD (R9C, ΔR14), AR (L39X) AD	Rare Rare, L39X and promotor variants

*AR* autosomal recessive

## *TTN* (Titin)

Titin, originally named “connectin” (Maruyama et al. 1977, 1976), appears as a long and flexible filament of about 1 µm in length and 3–4 nm-wide under the electron microscope (Maruyama et al. 1984; Trinick et al. 1984; Wang et al. 1984). Indeed, it is the largest known polypeptide found in nature, a single molecular spans from the Z-disk to M-band of the sarcomere. Titin is encoded by the gene *TTN* that is located on chromosome 2q31.2. The complete sequence of *TTN* contains 363 exons, and encodes up to 38,138 amino acid residues with a molecular weight of ~ 4.2 MDa (Bang et al. 2001).

Disease-causing missense pathogenic variants in *TTN* have been studied extensively in the context of skeletal muscle diseases, including hereditary myopathy with early respiratory failure (HMERF) and tibial muscular dystrophy or Limb Girdle Muscular Dystrophy (LGMD) (Hackman et al. 2002; Pollazzon et al. 2010; Toro et al. 2013). These AD myopathy-causing pathogenic variants are located in C-terminal regions of Titin. Recessive truncating and disruptive missense *TTN* pathogenic variants have also been linked to disease affecting both skeletal and cardiac muscles, such as early-onset myopathies with fatal cardiomyopathy (Carmignac et al. 2007; Chauveau et al. 2014a, b; Jungbluth and Gautel 2014).

Truncating variants in *TTN* are the most frequent genetic finding in idiopathic DCM being present in up to 25% of the cases (Herman et al. 2012) and are also found frequently in peri-partum cardiomyopathy (van Spaendonck-Zwarts et al. 2014). This observation was initially confounded by the appearance of *TTN* truncating variants in normal cohorts (up to 3%) (Roberts et al. 2015), but it is now evident that DCM associated *TTN* variants tend to cluster predominantly in the A-band portion of Titin, while variants found in controls tend to spare the A-band region and/or are in exons that have low usage in adult cardiac transcripts (Akinrinade et al. 2015a; Roberts et al. 2015; Schafer et al. 2017).

Autosomal dominant *TTN* missense pathogenic variants have been reported in various types of isolated CM (Gerull et al. 2002; Itoh-Satoh et al. 2002; Matsumoto et al. 2005; Peled et al. 2014; Satoh et al. 1999; Taylor et al. 2011). One of them, *TTN* W976R, is well supported by co-segregation within a large DCM family and functional data (Gerull et al. 2002; Gramlich et al. 2015; Hinson et al. 2015). Likewise, *TTN* A178D was identified in a family with features of left-ventricular non-compaction and DCM by a non-biased genetic approach. This pathogenic variant co-segregates with disease in the family and displays impaired function, i.e. reduced binding to the ligand telethonin in vitro (Hastings et al. 2016).

The causality for other *TTN* missense pathogenic variants is less clear, e.g. next generation sequencing identified large numbers of *TTN* missense variants in reference populations and in HCM (Lopes et al. 2013), but their relevance for the pathogenesis of HCM remains to be established. The challenge in clinical practice is that missense variants in *TTN* are common in normal population cohorts. One in three individuals carries a rare (i.e. < 0.01% allelic frequency) variant in *TTN*, but clearly only a small fraction of these are penetrant disease-causing pathogenic variants. Hence, *TTN* missense pathogenic variants are currently generally ignored when found in diagnostic sequencing (Akinrinade et al. 2015b; Lopes et al. 2013), however attempts are being made to classify them based on bioinformatics predictions (Haas et al. 2015; Helle and Parikh 2016; Laddach et al. 2017).

Expression of Titin is muscle-specific. Following Myosin and Actin, Titin is the third most abundant protein of striated muscle in vertebrates. As a result of alternative splicing of *TTN*, a number of Titin isoforms are expressed in different type of striated muscle tissues. In the heart, three major splicing isoforms of Titin have been identified: N2B, N2BA and fetal cardiac Titin. Both N2B and N2BA are isoforms of Titin expressed in adult cardiac muscle (Bang et al. 2001), whereas fetal cardiac Titin is expressed exclusively during development of the fetal heart (Lahmers et al. 2004). N2B is the shortest and stiffest isoform with the size of approximately 3.0 MDa, and is the predominant isoform of Titin expressed in rodent left ventricles (Bang et al. 2001). N2BA is the medium-sized isoform (3.3–3.5 MDa) with compliant stiffness that consists of both N2B and N2A elements, and contains an additional region of PEKV and Immuno-globulin like (Ig) domain elements. The ratio of N2BA to N2B isoforms varies among species and a higher ratio is found in larger animals (Cazorla et al. 2000). In contrast, fetal cardiac Titin is the largest and most compliant cardiac isoform of Titin with a molecular weight of 3.6–3.8 MDa (Lahmers et al. 2004).

A significant increase of N2BA to N2B ratio has been reported in DCM patients, hence affecting the passive tension within the sarcomere due to changes in stiffness (Nagueh et al. 2004). Altered expression ratio between these two Titin isoforms has also been described in a hypertensive myocardium rat model (Warren et al. 2003) and a canine heart failure model (Wu et al. 2002). Of note, RBM20 is an alternative splicing regulator, and pathogenic variants detected in DCM patients have been reported to disrupt protein function. This is associated with more compliant, larger Titin isoforms, which appears to drive the DCM phenotype (Guo et al. 2012). Moreover, this is supported by a RBM20 knockout rat model, which displays a DCM phenotype in the presence of more compliant, larger Titin isoforms.

As an intra-sarcomeric filament, Titin spans a half-sarcomere in length—Titin anchors the Z-disk at its N-terminus, crossing through the I-band and the A-band, towards the M-band at the C-terminus. Titin interacts with different sarcomeric proteins, for instance it binds to α-Actinin (Young et al. 1998), Telethonin (T-cap) (Gregorio et al. 1998; Mues et al. 1998) and may interact with Nebulin’s Src Homology 3 domain (Ma and Wang 2002) at the Z-disk. It binds Actin (Linke et al. 1997) and Obscurin (Young et al. 2001), and interacts with the Myosin filament through Myomesin at the M-line (Fukuzawa et al. 2008; van der Ven and Furst 1997). Cardiac Titin is considered a stable structural and flexible mechanical component of the myocardium, which prevents the overstretching of the sarcomere (Fürst and Gautel 1995; Maruyama 1997; Wang 1984). Titin plays an important role in regulating passive tension, an opposing force created during sarcomere stretching. With the elastic recoil properties largely derived from near the Z-disk towards the edge of the A-band, Titin acts like a “molecular spring” that contributes to the passive tension during cardiac relaxation (Helmes et al. 1999; Linke et al. 1999). The characteristics of this spring can further be modulated by phosphorylation events (reviewed in Hamdani et al. 2017; Kruger and Linke 2011).

In addition, Titin also acts as a scaffold protein for the thick filament proteins within the A-band region (Freiburg and Gautel 1996; Head et al. 2001). In this context, Titin has been suggested to function as a molecular ruler, controlling thick filament length (Bennett and Gautel 1996; Wang 1996; Whiting et al. 1989), however this concept is still controversially discussed (Granzier et al. 2014; Tskhovrebova et al. 2015).

Titin also contains binding sites that interact with several signalling proteins such as protease Calpain p94 (Kinbara et al. 1997), Muscle-specific Ring Finger Protein 1 (MURF-1) (Centner et al. 2001) and Four And A Half LIM Domains 2 (FHL2, also referred to as DRAL) (Lange et al. 2002). In addition, Titin features a serine/threonine kinase domain at the M-line (Gautel et al. 1993). This Titin kinase domain region is conformationally opened by mechanical strain (Puchner et al. 2008), then interacts with Nbr-1 and also recruits MURFs, proteins controlling protein turn-over in cardiomyocytes (Bogomolovas et al. 2014; Lange et al. 2005; Pizon et al. 2002). Moreover, Titin binds FHL1 and FHL2, both of which are implicated in mechano-responsive hypertrophic signalling (Lange et al. 2002; Raskin et al. 2012).

## *FHL1* (Four And A Half LIM Domains 1)

*FHL1* codes for a protein called Four And A Half LIM Domains 1 (FHL1). The gene is positioned on the X-chromosome (Xq26.3), and therefore pathogenic variants in this gene cause X-linked disease. *FHL1* was initially identified as a disease gene for skeletal muscle diseases, such as X-linked myopathy with postural muscle atrophy (Windpassinger et al. 2008), reducing body myopathy (Schessl et al. 2009), and Emery–Dreifuss muscular dystrophy (Gueneau et al. 2009). Particularly for Emery–Dreifuss muscular dystrophy cases, cardiac involvement is commonly observed, with conduction defects, arrhythmias, and hypertrophic cardiomyopathy. More recently, *FHL1* was also described as a disease gene for HCM, with or without skeletal muscle involvement (Friedrich et al. 2012; Hartmannova et al. 2013; Knoblauch et al. 2010).

FHL1 is a Titin-associated protein, with predominant expression in striated muscle tissues. As the name implies, it consists of four LIM domain and a fold resembling half a LIM domain (Lee et al. 1998). A LIM domain contains a cysteine rich consensus sequence [CX 2 CX 17–19 HX 2 CX 2 CX 2 CX 16–20 CX (2 C/H/D)] and comprises of two zinc fingers which coordinate one zinc ion each (Zheng and Zhao 2007). FHL1 is upregulated in human disease and experimental models of cardiomyopathy (Lu et al. 2012). In particular, the use of an alternative 5′ start site resulting in an “induced” iFHL1 transcript is associated with pathophysiological remodelling (Christodoulou et al. 2014). In the mouse model, inactivation of the gene has no baseline phenotype, however mice lacking FHL1 lack a response to pressure overload in the heart (Sheikh et al. 2008), suggesting that the protein is involved in mechano-signalling pathways. At the molecular level, FHL1 interferes with the phosphorylation of Titin N2B by Extracellular Signal Regulated-Kinase-2 (Erk2), thereby modulating Titin mechanics (Raskin et al. 2012).

Functional work on HCM-causing *FHL1* pathogenic variants suggests protein instability and loss of protein as the dominating contributor to disease (Friedrich et al. 2012). Moreover, FHL1 is discussed as a gender-specific modifier of disease severity in HCM patients, given its location on the X chromosome (Christodoulou et al. 2014).

## *CSRP3* (Muscle LIM Protein)

Muscle LIM Protein (MLP) was initially identified as a regulator of myogenesis in striated muscles (Arber et al. 1994). MLP is encoded by the gene Cysteine and Glycine-rich Protein 3 (*CSRP3*) on chromosome 11p15.1. Several pathogenic variants in *CSRP3* have been shown to cause cardiomyopathies with AD inheritance (Bos et al. 2006; Geier et al. 2003; Hershberger et al. 2008; Mohapatra et al. 2003). Almost all of the reported disease-causing pathogenic variants are located within the first 100 amino acids, no disease-causing variants been identified at the C-terminus (Vafiadaki et al. 2015). The increased availability of next generation sequencing data has helped to validate previously published pathogenic variants. One such variant, *CSRP3* p. W4R, described initially as a DCM-causing pathogenic variant (Knoll et al. 2002), has been re-classified as a benign polymorphism (Bos et al. 2006; Geier et al. 2008). Linkage analysis in a large German HCM pedigree led to identification of the C58G missense pathogenic variant in *CSRP3* (Geier et al. 2003). The MLP C58G mutant protein, when compared to MLP wildtype, was shown to be more susceptible to degradation in vitro. This supported findings that MLP levels in a cardiac biopsy were significantly reduced, up to 40% in a patient with a heterozygous MLP C58G pathogenic variant (Geier et al. 2008).

In addition to disease-causing pathogenic variants, MLP protein expression changes have been shown to be associated with cardiac disease. MLP was significantly reduced in failing hearts (Zolk et al. 2000), however, as MLP expression is variable in hearts, reduced expression cannot be used as a marker for heart failure.

MLP has been shown to be expressed exclusively in cardiomyocytes and in adult slow-twitch skeletal muscle cells (Arber and Caroni 1996; Schneider et al. 1999). MLP is a relatively small protein, consisting of 194 amino acids. The two LIM domains of MLP are followed by glycine-rich repeat regions, and separated by more than 50 residues. These LIM domains are also responsible for most of the MLP’s protein interactions, both structural and signalling related, in different regions of the cell (Arber and Caroni 1996; Kadrmas and Beckerle 2004; Schmeichel and Beckerle 1994, 1997; Weiskirchen et al. 1995). MLP has been shown to interact with Telethonin (T-cap) (Knoll et al. 2002), α-Actinin (Gehmlich et al. 2004; Louis et al. 1997) and Cofilin-2 (Papalouka et al. 2009) at the Z-disk. In vitro studies have also shown additional binding partners for MLP. For example, MLP can bind to itself (Zolk et al. 2000), it associates with proteins at the costamere (including, Zyxin, Integrin Linked Kinase, and β1-Spectrin) (Flick and Konieczny 2000; Postel et al. 2008; Zolk et al. 2000) and the Nebulin-related Anchoring Protein (N-RAP) (Ehler et al. 2001) at the intercalated disk. MLP also interacts with the nuclear transcription factors MyoD, Myogenin, and Myogenic Regulatory Factor 4 (MRF4) (Kong et al. 1997). MLP’s interactions with these transcription factors, and the presence of predicted nuclear localization signal suggested that MLP function is regulated by translocation between nucleus and cytoplasm (Boateng et al. 2009). There is conflicting information about MLP’s localisation within cardiac cells. MLP has been proposed to be a sarcomere protein located at the Z-disk, I-band, M-line, or at the cell membrane (Arber and Caroni 1996; Arber et al. 1997; Flick and Konieczny 2000; Henderson et al. 2003; Knoll et al. 2010). However, it has also been reported to be a non-sarcomeric protein, with diffuse cytoplasmic expression (Geier et al. 2008).

MLP Knockout (KO) mice were one of the first published models for dilated cardiomyopathy, with a molecular activation of hypertrophic signalling cascades (Arber et al. 1997). MLP KO cardiomyocytes exhibit cytoarchitecture perturbations including disrupted myofibrillar assembly, abnormal alignment of Z-disks and marked fibrosis (Arber et al. 1997). Aberrations at the intercalated discs were observed in these mice, with upregulation of proteins including N-RAP, β-Catenin, Vinculin and plakoglobin, along with upregulation of adherens junctions and downregulation of the gap junction protein Connexin-43 (Ehler et al. 2001). Other studies have also highlighted that loss of MLP leads to perturbation in intracellular calcium handling and excitation–contraction coupling and that a double knockout of MLP and Phospholamban, which regulates sarcoplasmic reticulum calcium intake, rescues the DCM phenotype (Esposito et al. 2000; Kemecsei et al. 2010; Kuhn et al. 2012; Minamisawa et al. 1999, Su et al. 2001).

MLP KO mice are born in Mendelian frequencies, dismissing an indispensable role in embryonic development, however, the protein is thought to be essential for adaptation of the heart to increased hemodynamic stress post birth (Buyandelger et al. 2011). MLP deficiency resulted in loss of passive elasticity in isolated papillary muscles from neonatal and perinatal cardiomyocytes. This has been suggested as a contributing factor to development of diastolic dysfunction and eventual heart failure in these animals. Increased stiffness of cardiomyocytes was also demonstrated by Omens and colleagues in their study performed on hearts from 2-week-old MLP-deficient animals (Omens et al. 2002). The underlying molecular mechanism of this effect, however, is still poorly understood. Prolonged mechanical stress results in maladaptive changes in the cardiomyocytes leading to hypertrophy and eventual heart failure. These observed changes in elasticity, combined with findings that mechanical stimulation failed to stimulate BNP transcription in MLP KO cardiomyocytes, led to the proposal that MLP is part of cardiac stretch sensor complex, along with Titin and Telethonin (Knoll et al. 2002). These suggestions were made considering the findings that MLP was localised to Z-disk. However, more recent findings of MLP’s cytoplasmic localisation (Geier et al. 2008) makes it unlikely that a non-sarcomeric protein such as MLP can be a stress sensor for cardiomyocytes. It is likely that MLP is rather involved in downstream signalling pathways.

MLP heterozygous KO mice (MLP +/−) show no overt phenotype under normal conditions. Compared to WT animals, these mice present with more left ventricular dilation and systolic dysfunction and decreased survival after myocardial infarction; this is associated with a supressed pro-hypertrophic Calcineurin-Nuclear Factor of Activated T-cells (NFAT) signalling pathway (Heineke et al. 2005), again underlining MLP’s role in hypertrophic signalling cascades. Moreover, MLP protein levels have been shown to increase during stress such as aortic banding in wild-type mice (Kuhn et al. 2012). However, overexpression of MLP does not confer any protection to the heart in response to pathological stress such as transverse aortic constriction or chronic infusion of angiotensin-II (Kuhn et al. 2012).

Further, the novel function of MLP as an endogenous inhibitor of Protein Kinase C α (PKCα) in the heart has been elucidated (Lange et al. 2016): Aberrant PKCα signalling in the heart has been shown to cause remodelling and pathological growth of the heart. In the absence of MLP the expression of adapter protein CARP was increased, which led to recruitment of PKCα at the intercalated disc. The absence of CARP reduces PKCα signalling at the intercalated disc, which is why mice lacking both MLP and CARP develop normally and show no signs of DCM (Lange et al. 2016).

## *FLNC* (Filamin C)

Filamin C is encoded by *FLNC* on chromosome 7q32.1. It is an established disease gene for skeletal muscle disease, causing protein aggregation myofibrillar myopathy (MFM) (Vorgerd et al. 2005) or distal myopathy (Duff et al. 2011). Cardiac involvement has been described for approximately one-third of MFM cases (Kley et al. 2007; Vorgerd et al. 2005). More recently, pathogenic variants in Filamin C were reported in families with familial HCM without skeletal muscle involvement (Valdes-Mas et al. 2014). The majority of the reported putative pathogenic variants were missense changes. In addition, two further missense pathogenic variants were reported in individuals with RCM (Brodehl et al. 2016). Prompted by these findings, screening was expanded onto other types of CMs and subsequently pathogenic variants in *FLNC* were also associated with DCM and ARVC (Ortiz-Genga et al. 2016). It now emerges that missense pathogenic variants tend to cause HCM or RCM (Brodehl et al. 2016; Gomez et al. 2017), while nonsense and truncation pathogenic variants cause DCM or ARVC (Begay et al. 2016; Janin et al. 2017; Ortiz-Genga et al. 2016).

Filamin C is highly expressed in muscle tissues. It belongs to the family of three Filamin proteins (A, B and C), all characterised by the same modular blueprint (Razinia et al. 2012): at the N-terminus, two calponin- homology domains form an Actin-binding interface, which is followed by 24 Ig-domains. The last of these domains (d24) mediates dimerization of the protein (Himmel et al. 2003; Sjekloca et al. 2007). As a result of this Y-shaped structure, Filamins are Actin-cross linking proteins.

Unique for Filamin C is a striated-muscle specific 80 amino acid long insertion in Ig-domain 20, which mediates interactions to ligands such as e.g. Myotilin (van der Ven et al. 2000), Myopodin (Linnemann et al. 2010), Xin and XIPR2 (van der Ven et al. 2006) and aciculin (Molt et al. 2014). Of note, many of these proteins have striated-muscle specific expression (Myotilin, Myopodin, Xin, XIPR2) and are thought to have a crucial role for the organisation and integrity of skeletal and/or cardiac tissue. For example, Myotilin is a known disease gene for LGMD (Salmikangas et al. 1999) and MFM (Selcen and Engel 2004) and deletion of Xin proteins in mouse models leads to either mild cardiac abnormalities (Otten et al. 2010) or cardiac hypertrophy and electrophysiological changes (Chan et al. 2011; Gustafson-Wagner et al. 2007).

Many of the other Ig-domains have also been found to mediate interactions with ligands (reviewed in van der Flier and Sonnenberg 2001; Zhou et al. 2007) and the protein’s function are modulated by protein phosphorylation events (Murray et al. 2004; Reimann et al. 2017; Sequea et al. 2013).

Like the other members of the Filamin family, Filamin C modulates Actin dynamics. It plays important roles in myofibrillogenesis (Chiang et al. 2000; Dalkilic et al. 2006) by acting in concert with its binding partners Xin, XIRP2 and Aciculin (Molt et al. 2014). A mouse model with genetic inactivation of Filamin C highlights the protein’s crucial role for muscle function; Filamin C deficient mice die at birth due to respiratory failure and have underdeveloped skeletal muscles (Dalkilic et al. 2006).

In mature striated muscle, Filamin C is found at the periphery of the Z-disks, linking sarcomeric Actin structures to the cytoskeleton (Gontier et al. 2005), and at the intercalated disk, a structure which links neighbouring cardiomyocytes to each other. Beyond its structural roles, Filamin C acts as a signalling hub and is an active player in the repair of myofibrillar damage in cardiomyocytes (Leber et al. 2016). Based on its homology and structural similarity with Filamin A, mechano-sensing functions have been postulated (Razinia et al. 2012). The Ig-domains 20–21 of Filamin A have been shown to be in a closed conformation that opens upon mechanical stretch and is subsequently accessible for ligands (Chen et al. 2013; Seppala et al. 2015), thereby providing a molecular basis for how altered mechanical load can trigger downstream signalling events, such as myofibrillar repair.

Filamin C has been identified as a target of chaperone assisted selective autophagy (CASA) (Arndt et al. 2010; Ulbricht et al. 2015). Upon mechanical stress, damaged components of the Z-disk such as Filamin C will be released in a chaperone BAG3-mediated process and targeted for degradation by the autophagosome. This process seems de-regulated in skeletal muscle diseases (especially MFM) when aggregates of mutant Filamin C proteins form (Kley et al. 2013b). These aggregates aberrantly recruit myofibrillar components and hence deplete them from the myofilament (Kley et al. 2013a). Moreover, the CASA mechanism and subsequent autophagy are impaired in the presence of these protein aggregates (Ruparelia et al. 2016).

The patho-mechanisms of Filamin C-related cardiomyopathies are less clear. For DCM, nonsense and truncating pathogenic variants appear to dominate. However, why these pathogenic variants cause pure cardiac disease, mostly without skeletal disease involvement, is still unclear. The absence of Filamin C protein aggregates in the myocardium of DCM patients with Filamin C pathogenic variants is a valuable observation (Ortiz-Genga et al. 2016) and it could be speculated that a loss of function mechanism prevails. In contrast, for some (but not all) HCM/RCM patients with Filamin C pathogenic variants investigated, protein aggregation has been observed in vivo and in vitro (Brodehl et al. 2016; Valdes-Mas et al. 2014). It is currently speculated that depending on the positions of the missense pathogenic variant in the protein, these mutants may cause disease through different modes of action (Gomez et al. 2017).

While Filamin C is now recognised as an important disease gene for cardiomyopathies, future functional work, including the generation of model systems and organisms, is needed to gain insights into the detailed pathophysiology of cardiomyopathies.

## *PLN* (Phospholamban)

Phospholamban is encoded by *PLN* on chromosome 6q22.31. It is a rare, but well established disease gene for DCM, with several disease-causing missense pathogenic variants identified in familial cohorts. A causative role for *PLN* R9C in DCM is evidenced by co-segregation in a large 4 generation family affected by DCM and heart failure (Schmitt et al. 2003). Additionally, the L39X pathogenic variant was identified in another large family, resulting in left ventricular hypertrophy in heterozygous carriers and DCM in homozygous carriers in the absence of detectable Phospholamban protein (Haghighi et al. 2003). Interestingly, the heterozygous L39X pathogenic variant has also been found in patients with HCM (Chiu et al. 2007; Landstrom et al. 2011). Another pathogenic variant supported by co-segregation in a large family with DCM is the deletion of arginine 14 (Haghighi et al. 2006), which has also been found in other, unrelated individuals and/or families affected by DCM (DeWitt et al. 2006; Posch et al. 2009). In addition, two pathogenic variants in the promoter region of *PLN* have been associated with HCM (Medin et al. 2007; Minamisawa et al. 2003), with functional studies showing opposing effects on promotor activity.

Functionally, Phospholamban associates with the Sarcoplasmic Reticulum Calcium ATPase (SERCA2a) (Verboomen et al. 1992) and acts to negatively regulate intracellular calcium removal through direct inhibition of SERCA-mediated calcium uptake into the sarcoplasmic reticulum. Under basal conditions, Phospholamban exists in equilibrium between its monomeric and pentameric form (Fujii et al. 1989), with phosphorylation demonstrated to stabilize the pentameric structure and reduce the affinity of Phospholamban to SERCA2a (Hou et al. 2008). Phosphorylation of serine 16 by Protein Kinase A (PKA) and threonine 17 by Calcium/Calmodulin-dependent Protein Kinase occur in response to beta-adrenergic stimulation (Wegener et al. 1989). Both phosphorylation events release SERCA2a inhibition, thereby increasing SERCA2a’s transport of calcium from the cytosol into the lumen of the sarcoplasmic reticulum during diastole.

Ablation of Phospholamban in mice leads to enhanced myocardial performance (Luo et al. 1994), equivalent to that of wildtype hearts with fully activated by beta-adrenergic stimulation. As such, ablation of Phospholamban has subsequently been used as an experimental approach to improve cardiac function in rodent models of heart failure (Kaneko et al. 2016; Mazzocchi et al. 2016; Minamisawa et al. 1999; Tsuji et al. 2009; Zhang et al. 2010).

Mouse models for DCM-associated *PLN* pathogenic variants provide sufficient evidence to support a disease-causing role of *PLN* pathogenic variant in cardiac disease. Transgenic mice carrying the deletion of arginine 14 in Phospholamban die between 2 and 16 weeks of age due to ventricular dilatation and heart failure (Haghighi et al. 2006). At the molecular level, the mutant protein fails to inhibit SERCA2a due to a lack of physical interaction (Haghighi et al. 2012), and instead is mis-localises to the sarcolemmal Na/K-ATPase where it activates its pump function. Transgenic mice expressing the R9C pathogenic variant are also characterised by heart failure and premature death (Schmitt et al. 2003). In these mice, the mutant protein traps PKA and thereby blocks phosphorylation of wildtype Phospholamban. Molecular studies have shown that R9C stabilises the pentameric form of Phospholamban due to disulfide bond formation, preventing phosphorylation by PKA and interaction with SERCA2a (Ha et al. 2011). The R9C transgenic mice have subsequently been used to study disease progression in DCM on the transcriptome and proteome level (Burke et al. 2016; Kuzmanov et al. 2016).

Though pathogenic variants in *PLN* are rare, findings from *PLN* mutant carriers and mouse models demonstrate that changes in calcium handling in the presence of Phospholamban pathogenic variants, secondary to perturbations in SERCA2a activity, are sufficient to cause cardiomyopathy.

## Conclusions

We have demonstrated with the examples of Titin, FHL1, MLP/Csrp3, Filamin C and Phospholamban discussed here, that there are disease genes for cardiomyopathies beyond the “classical” genes coding for proteins with exclusively structural roles in the sarcomere or the cytoskeleton. It emerges that signalling pathways, often involved in the detection and adaptation to increased load in the normal heart (e.g. acutely upon sympathetic stimulation or more chronically in the presence of hypertension), can be disturbed by pathogenic variants in the genes discussed here and that these chronic disturbances of signalling pathways result in cardiomyopathic changes over a long period of time (often decades).

Our understanding of disease mechanisms lags behind the genetic findings and future work will need to elucidate how pathogenic variants in these genes cause cardiomyopathies. In addition to biochemical in vitro experiments, model organisms such as zebrafish (Asnani and Peterson 2014; Wilkinson et al. 2014) and mice (Camacho et al. 2016) can help gain insight into the complex changes at whole organ level. A novel, emerging technology to model disease in vitro are human induced pluripotent stem cell derived cardiomyocytes, allowing the generation of patient-derived human cardiomyocytes with a specific genetic pathogenic variant. Together with recent advances in genome-editing technologies, induced pluripotent stem cell derived cardiomyocytes have emerged as a powerful tool to explore patho-mechanisms of cardiomyopathies (reviewed in Giacomelli et al. 2017; Sallam et al. 2014).

With exception of *TTN* truncating variants in DCM, the pathogenic variants in the genes discussed here are individually rare, but collectively they contribute to an estimated 3% of cases in cardiomyopathy cohorts. With the wide-spread application of high-throughput sequencing techniques in the clinical practice, these disease genes will be increasingly interrogated. The challenge remains to confidently assign or disregard a causative role of a variant for the cardiomyopathy phenotype observed in an individual—a classification as “variant of unknown significance” is not helpful e.g. for predictive testing in family members. New bio-informatics approaches in combination with simple, high throughput wet-lab approaches will need to be developed to tackle this challenge.
